# Influences of 27-Gauge Vitrectomy on Corneal Topographic Conditions

**DOI:** 10.1155/2017/8320909

**Published:** 2017-08-27

**Authors:** Takafumi Hirashima, Takao Utsumi, Miou Hirose, Hideyasu Oh

**Affiliations:** Department of Ophthalmology, Hyogo Prefectural Amagasaki General Medical Center, Higashinanima-cho 2-17-77, Amagasaki, Hyogo 660-8550, Japan

## Abstract

**Purpose:**

To evaluate the influences of 27-gauge vitrectomy on corneal topographic conditions.

**Method:**

Fifty-six eyes of 56 patients undergoing 27-gauge vitrectomy were retrospectively studied. Twenty-three eyes with epiretinal membrane (ERM), 23 eyes with macular hole (MH), and 10 eyes with proliferative diabetic retinopathy (PDR) were included. Forty-five of the 56 eyes underwent 27-gauge phacovitrectomy (group 1), and the remaining 11 eyes underwent 27-gauge vitrectomy alone (group 2). Corneal topography was obtained with a wave-front analyzer preoperatively and at 1 and 3 months postoperatively. The corneal topographic parameters evaluated were the average corneal power, regular astigmatism, spherical aberration, and higher-order aberration (HOA).

**Results:**

In between-group analyses of groups 1 and 2, no significant differences were observed regarding the changes of the 4 parameters from the baseline to 1 and 3 months postoperatively. No significant differences in the changes of all parameters from the baseline to 1 and 3 months postoperatively were also observed between MH group and the other two groups. A significant difference in the change of HOA from the baseline to 1 month postoperatively was observed between ERM and PDR group however, the difference disappeared at 3 months.

**Conclusion:**

27-gauge vitrectomy did not induce substantial changes in the corneal topographic conditions.

## 1. Introduction

The modern vitrectomy surgery has been refined and improved by continuous development of smaller instruments and greater functionality. Recently, the 27-gauge vitrectomy has been introduced as a less invasive surgery. This was made possible also by the development of powerful light sources, ultrahigh-speed cutter, and wide-angle viewing systems [1–3]. There are several advantages of sutureless small-guage vitrectomy such as less traumatic sclera manipulation, less intraocular inflammation, and reduced visual recovery time [4–6]. The newly developed 27-gauge vitrectomy may exert less influence on corneal topography after surgery.

Previous studies have reported that conventional 20-guage vitrectomy induced significant changes in corneal topography [7–9]. The surgically induced astigmatism (SIA) is transient and returned to baseline level by 4 months after surgery [8, 10]. In contrast, Yanyali et al. [11] reported that there was no significant change in regular astigmatism (RA) at one week and one month after 25-gauge vitrectomy. In the study by Okamoto et al. [12], for RA and higher-order aberration (HOA), the 20-guage surgery showed significantly greater surgically induced changes than the 25-gauge vitrectomy. Park et al. [13] also reported that the SIA of 23-gauge phacovitrectomy was significantly less than that of the conventional 20-guage phacovitrectomy during 3 months after surgery. The 25- or 23-guage vitrectomy has simplified the vitrectomy procedure and may have less influence on corneal topography than the conventional 20-guage vitrectomy. However, the influences of 27-gauge vitrectomy on corneal topography still remain to be elucidated. In this report, we first conducted a study regarding the corneal topographical changes after 27-gauge vitrectomy alone or phacovitrectomy.

## 2. Methods

### 2.1. Study Design

This was a retrospective study. This study was performed in a single center at the Hyogo Prefectural Amagasaki Hospital, Japan, between December 2012 and July 2014. This study was approved by the local Institutional Review Board and Ethics Committee and conformed to the Declaration of Helsinki. Informed consent was obtained from all patients.

### 2.2. Patients

Fifty-six eyes of 56 consecutive patients undergoing 27-gauge vitrectomy at the Hyogo Prefectural Amagasaki Hospital between December 2012 and July 2014 were included in this study. Forty-five of the 56 eyes (80.3%) underwent phacovitrectomy (group 1), and the remaining 11 eyes underwent 27-gauge vitrectomy alone (group 2). Nine of the 11 eyes had previously undergone cataract surgery. The other 2 eyes underwent lens-sparing vitrectomy. Indications for vitrectomy were epiretinal membrane (18 eyes in group 1 and 5 eyes in group 2), macular hole (19 eyes in group 1 and 4 eyes in group 2), and proliferative diabetic retinopathy (PDR) (8 eyes in group 1 and 2 eyes in group 2). Six of the 10 eyes in PDR had persistent vitreous hemorrhage, and 3 eyes had tractional retinal detachment (TRD). One eye had both persistent vitreous hemorrhage and TRD.

Eyes were excluded if there was a pre-existing corneal pathology such as corneal dystrophy, corneal ectasia and corneal infection, severe refractive error (more than −6 diopters), or complicated vitreoretinal diseases such as trauma-induced retinopathy, retinal detachment with giant retinal tears since extensive vitreous base shaving with scleral indentation is inevitable.

### 2.3. Surgical Procedures

All eyes underwent vitrectomy using the Accurus Vitrectomy System (Alcon Laboratories, Fort Worth, TX, USA) in combination with a cut rate booster, UST (ultraspeed transformer; DORC, Zuidland, Holland). Three 27-guage cannulas (DORC) were inserted transconjunctivally into the eye with a designed blunt inserter (DORC). These 3 microcannulas were placed 4.0 mm posterior to the limbus at 2, 8, and 10 o'clock (right eye) or at 2, 4, and 10 o'clock (left eye). We used a twin duty cycle vitreous cutter (DORC) which can increase the actual cut rate 2-fold compared to a conventional cutter. The vitreous cut rate is set at 6000 cuts per minute, and the aspiration pressure is set at the maximum of 600 mmHg. We also used a light probe (Shielded TotalView; DORC) connected to the Xeon Brightstar (DORC) and did not use additional lights including a twin light chandelier. In a total of 56 eyes, core vitrectomy was performed, and the posterior hyaloid separation was created if not present. Using a wide-angle view system (Resight 500), we performed moderate not but extensive vitreous base shaving as much as possible without scleral indentation in PDR cases as well as in the two other diseases. In both ERM and MH groups, the internal limiting membrane peeling was performed by using Brilliant Blue G staining. Fluid-air exchange followed by 20% SF6 gas injection was combined only in MH group (23 cases). We performed endolaser photocoagulation in PDR group (10 cases). At the end of surgery, all cannulas were removed, and neither the conjunctival nor the scleral wounds required suture in all of the 56 cases.

Forty-five of 56 eyes underwent phacoemulsification using the Infinity Vision System (Alcon Laboratories, Fort Worth, TX, USA) before vitrectomy. The procedure was started with a 2.2 mm corneoscleral incision at 12 o'clock. Two paracentesises were created at 2 and 10 o'clock. After phacoemulsification and irrigation/aspiration, the capsular bag was expanded with viscoelastic materials and then a foldable 6.0 mm intraocular lens (W-60, Santen) was implanted in the bag.

At the completion of surgery, the dexamethasone (1.65 mg) was injected subconjunctivally. The single surgeon (H.O.) performed all operations.

### 2.4. Measurement of Corneal Topography

**Figure 1:**
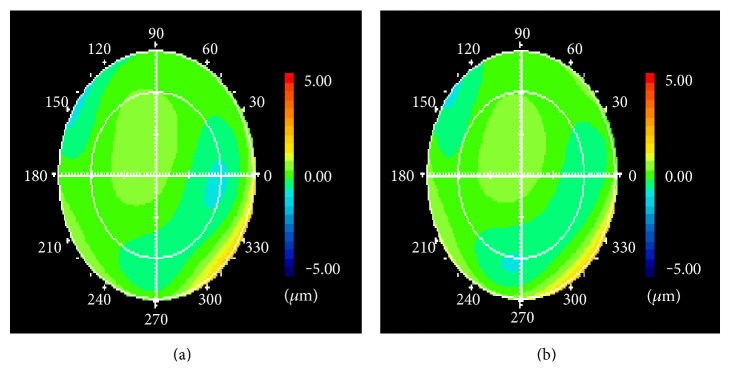
Preoperarive (a) and postoperative 1-month (b) corneal topographic map. The distribution of the color map at 1 month after surgery for epiretinal membrane was almost the same as preoperative.

Corneal topography was measured by a wave-front analyzer (KR-1W® Topcon) preoperatively and also at 1 and 3 months postoperatively (Figure 1). The corneal topographic parameters studied included the average corneal power (CP), regular astigmatism (RA), spherical aberration (SA), and higher-order aberration (HOA).

The statistically significant differences between two groups were evaluated using a Mann–Whitney test. For within-group analysis, a Wilcoxon test was employed. A *P* value < 0.05 was considered to be statistically significant. The best-corrected visual acuity (BCVA) was converted to the logarithm of the minimum angle of resolution (logMAR) before analysis.

## 3. Results

Of the 56 eyes, 32 were from women and 24 were from men. The mean age of the patients was 66.6 ± 8.4 years (range, 32 to 81 years). The mean follow-up was 8.6 ± 3.6 months (range, 4 to 18 months).

### 3.1. Surgical Outcome

In the ERM group, the BCVA significantly improved from 0.39 to 0.17 at 1 month and 0.11 at 3 months after the surgery (*P* < 0.05). Removal of ERM was achieved in all of the 23 eyes of the ERM group. In the MH group, the BCVA also significantly improved from 0.57 to 0.33 at 1 month and 0.24 at 3 months after the surgery (*P* < 0.05), and macular hole was successfully closed in cases. In the PDR group, the BCVA significantly improved from 1.12 to 0.45 at 1 month and 0.42 at 3 months after the surgery (*P* < 0.05). Retinal reattachment was obtained in eyes with preoperative tractional retinal detachment, and recurrence of vitreous hemorrhage or retinal detachment was not observed during the 6 months follow-up period. In each group, the formation of a subconjunctival bleb indicating leakage was not seen after each sclerotomy site was pressed with a sterile cotton-tipped applicator for about 10 seconds.

In all groups, intraoperative complications such as rupture of the posterior capsule, iatrogenic tears, and/or retinal detachment were not observed. As to the postoperative complications, there were 2 eyes that showed increased intraocular pressure (>21 mmHg), but all returned to normal conditions within 1 week and no eyes required additional surgery for lowering the pressure. Other postoperative complications including ocular hypotony were not observed for the entire follow-up period.

### 3.2. Phacovitrectomy versus Vitrectomy Alone

The corneal topographic parameters of both of groups 1 and 2 are listed in Table 1. In between-group analysis, no significant differences were observed regarding the changes of the 4 parameters from the baseline to 1 and 3 months. Similarly, in within-group analysis, all of the 4 parameters at 1 and 3 months did not change significantly compared to those at baseline in both of groups 1 and 2.

### 3.3. ERM Group versus MH Group versus PDR Group

**Figure 2:**
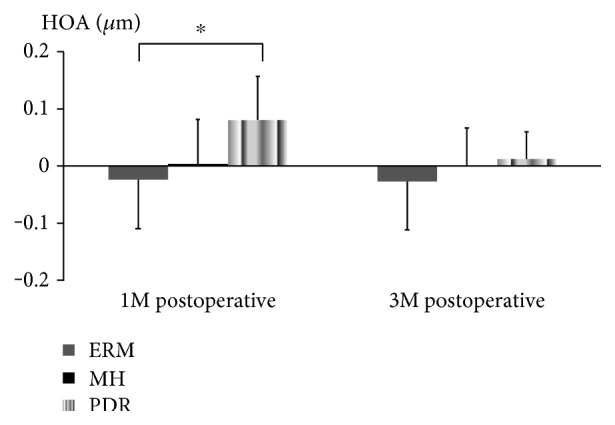
With regard to the change of higher-order aberration (HOA), there was a significant difference between epiretinal membrane (ERM) group and proliferative diabetic retinopathy (PDR) group at 1 month (M) after the surgery (*P* < 0.05). No significant differences were observed between macular hole (MH) group and the other two groups. ^∗^*P* < 0.05.

The corneal topographic parameters of the ERM, MH, and PDR group are listed in Table 2. In between-group analysis of the MH and ERM groups, there were no significant differences in parameters between the values at 1 or 3 months and baseline. Similarly, in between-group analysis of MH and PDR group, no significant differences in parameters between the values at 1 or 3 months and baseline were observed. In contrast, in between-group analysis of ERM and PDR group, there was a significant difference in the HOA between the value at 1 month and baseline (*P* < 0.05) (Figure 2). For the other 3 parameters, there were no significant differences between the two groups at 1 month after the surgery. In addition, there were no significant differences in the change of all parameters from the baseline to 3 months between the two groups. In within-group analysis, the 4 corneal topographic parameters remained stable up to 3 months after the surgery in all groups (*P* > 0.5 for all parameters).

## 4. Discussion

In this study, we demonstrated that both 27-gauge vitrectomy alone and phacovitrectomy do not induce any significant changes in corneal topography. It is consistent with the view that 27-gauge vitrectomy induce little influence on corneal topography as with 25- or 23-guage vitrectomy.

The presence of cataracts often necessitates a second operation soon after vitrectomy if lens extraction is not performed simultaneously with the vitrectomy. Moreover, vitrectomy itself causes cataracts to progress. Thus, treating cataract and vitreoretinal diseases in a combined one-step microincision phacovitrectomy is an effective and well-tolerated method and is becoming one of the common procedures [14–16]. In our study, 45 of the 56 eyes (80.3%) underwent phacovitrectomy. Phacovitrectomy eliminates the need for a second operation, and also offers potential for better surgical outcomes partially due to the improved access to the retinal periphery [16, 17]. With regard to influences on corneal topography, there were no significant differences between phacovitrectomy and vitrectomy alone, and thus further supports the usefulness of the combined procedure.

HOAs are part of the refractive errors which are not by standard spherocylindrical correction and can deteriorate the quality of the retina [18]. Recently, they have been considered to be important indices in the field of quality of vision [19, 20]. HOAs mainly consist of both comatic and spherical aberration and especially, SA has been found to be linked to contrast sensitivity. When SA increases, contrast sensitivity decreases and greater amounts of halos and glare are induced [21]. Corneal positive SA is reported to be approximately 0.27 *μ*m, which is consistent with the overall mean SA in our study (0.27 ± 0.08 *μ*m) [22, 23]. In each of ERM, MH, and PDR group, the corneal topographic parameters, including both SA and HOA, remained stable for up to 3 months after the surgery. In contrast, in between-group analysis of ERM and PDR group, significant difference in the HOA between the value at 1 month and baseline was observed. This may be due to, at least partly, the fact that in PDR cases additional procedures like endolaser photocoagulation and removal of membranes located outside of the posterior pole are often required and thus may influence the wound and the cornea. The changes of the HOA from the baseline to 1 month in PDR group with or without TRD were 0.16 ± 0.14 *μ*m and 0.03 ± 0.01 *μ*m, respectively, (*P* = .30).

Overall surgical outcomes in our study were encouraging, as with the recent report of 27-gauge vitrectomy [24]. Anatomic recovery was achieved in all of the eyes in each of ERM, MH, and PDR group after the initial surgery. Although transient ocular hypertension existed in 2 cases postoperatively, it spontaneously recovered to normal range within 1 week. Postoperative hypotony was not observed in all cases during the follow-up time. In conclusion, neither 27-gauge vitrectomy alone nor 27-guage phacovitrectomy induces substantial changes in the corneal topographic conditions in total. Considering these results and a major postoperative complication of vitrectomy, cataract progression, 27-gauge phacovitrectomy might be a safe and effective option especially in cases with preoperative substantial cataract. Limitations of this study were the small sample size and the lack of comparing groups such as the 25- or 23-guage vitrectomy. A prospective randomized patient study is warranted to compare influences of 27-gauge vitrectomy and 25- or 23-guage vitrectomy on postoperative corneal topographic conditions.

## Figures and Tables

**Table 1:** The corneal topographic parameters in groups 1 and 2. No significant differences were observed regarding the changes of the 4 parameters from the baseline to 1 and 3 months between the two groups.

	Group 1 (*n* = 11)			Group 2 (*n* = 45)		
	Preoperative	Postoperative 1M	Postoperative 3M	Preoperative	Postoperative 1M	Postoperative 3M
Average corneal power (D)	44.5 ± 1.46	44.6 ± 1.46^†^	44.6 ± 1.49^†^	44.6 ± 1.29	44.7 ± 1.20^†^	44.8 ± 1.18^†^
Regular astigmatism (D)	−1.47 ± 0.51	−1.06 ± 0.53^†^	−1.24 ± 0.48^†^	−1.18 ± 0.67	−1.09 ± 0.65^†^	−1.10 ± 0.55^†^
Spherical aberration (*μ*m)	0.27 ± 0.06	0.26 ± 0.06^†^	0.25 ± 0.07^†^	0.27 ± 0.09	0.27 ± 0.07^†^	0.26 ± 0.07^†^
Higher-order aberration (*μ*m)	0.18 ± 0.10	0.18 ± 0.08^†^	0.16 ± 0.07^†^	0.19 ± 0.08	0.19 ± 0.09^†^	0.18 ± 0.07^†^

M = month; D = diopter; ^†^NS versus preoperative.

**Table 2:** The corneal topographic parameters in epiretinal membrane (ERM) and macular hole (MH) and proliferative diabetic retinopathy (PDR) group. In all groups, the 4 parameters remained stable up to 3 months after the surgery.

	ERM group (*n* = 23)			MH group (*n* = 23)			PDR group (*n* = 10)		
	Preoperative	Postoperative 1M	Postoperative 3M	Preoperative	Postoperative 1M	Postoperative 3M	Preoperative	Postoperative 1M	Postoperative 3M
Average corneal power (D)	44.5 ± 1.09	44.5 ± 1.07^†^	44.6 ± 1.04^†^	44.8 ± 1.41	44.9 ± 1.28^†^	45.0 ± 1.36^†^	44.4 ± 1.58	44.6 ± 1.53^†^	44.6 ± 1.36^†^
Regular astigmatism (D)	−1.18 ± 0.56	−1.13 ± 0.58^†^	−1.19 ± 0.53^†^	−1.26 ± 0.79	−1.07 ± 0.72^†^	−1.07 ± 0.60^†^	−1.30 ± 0.50	−1.01 ± 0.54^†^	−1.12 ± 0.55^†^
Spherical aberration (*μ*m)	0.25 ± 0.07	0.26 ± 0.04^†^	0.26 ± 0.05^†^	0.27 ± 0.10	0.27 ± 0.09^†^	0.27 ± 0.09^†^	0.30 ± 0.08	0.30 ± 0.08^†^	0.27 ± 0.08^†^
Higher-order aberration (*μ*m)	0.20 ± 0.09	0.17 ± 0.06^†^	0.17 ± 0.06^†^	0.17 ± 0.08	0.17 ± 0.05^†^	0.18 ± 0.08^†^	0.21 ± 0.07	0.29 ± 0.14^†^	0.22 ± 0.06^†^

M = month; D = diopter; ^†^NS versus preoperative.
